# Mitochondrial gene sequence variants in children with severe malaria anaemia with or without lactic acidosis: a case control study

**DOI:** 10.1186/s12936-018-2618-5

**Published:** 2018-12-13

**Authors:** Casey Fowler, Christine Cserti-Gazdewich, Aggrey Dhabangi, Charles Musoke, Himanshu Sharma, Sami S. Amr, Walter Dzik

**Affiliations:** 1 Translational Genomics Core Laboratory, Partners Personalized Medicine, Boston, MA USA; 2 Department of Pathobiology and Laboratory Medicine, University of Toronto, Toronto, Canada; 3 Department of Pediatrics and Childhealth, Makerere University, Kampala, Uganda; 4 Department of Medicine, Makerere University, Kampala, Uganda; 5 Department of Pathology and Medicine, Massachusetts General Hospital, Boston, MA USA

**Keywords:** Mitochondria, Malaria, Lactic acidosis, Anemia, Tissue oxygenation, DNA sequence

## Abstract

**Background:**

Evolutionary pressure by *Plasmodium falciparum* malaria is known to have favoured a large number of human gene adaptations, but there is surprisingly little investigation of the effect of malaria on human mitochondrial sequence variation. *Plasmodium falciparum* infection can cause severe malaria anaemia (SMA) with insufficient tissue oxygenation, lactic acidosis and death. Despite equal degrees of severe anaemia, some individuals develop lactic acidosis while others do not. A case–control study design was used to investigate whether differences in host mitochondrial gene sequences were associated with lactic acidosis in SMA. Full mitochondrial sequences were obtained from 36 subjects with SMA complicated by lactic acidosis and 37 subjects with SMA without lactic acidosis. The two groups were matched for age, sex, and degree of anaemia.

**Results:**

Compared with the reference sequence, a median of 60 nucleotide variants per individual (interquartile range 4–91) was found, with an average frequency of 3.97 variants per 1000 nucleotides. The frequency and distribution of non-synonymous DNA variants in genes associated with oxidative phosphorylation were not statistically different between the two groups. Non-synonymous variants predicted to have the most disruptive effect on proteins responsible for oxidative phosphorylation were present at a similar frequency in both groups.

**Conclusions:**

Lactic acidosis in SMA does not appear to be consistently associated with the high prevalence of any mitochondrial gene variant.

## Background

Malaria results in the death of hundreds of thousands of children each year [1]. Genetic adaptations affecting host susceptibility play a major role in survival from malaria [2, 3]. Because most deaths from *Plasmodium falciparum* malaria occur in children prior to puberty, malaria has been recognized as “*the strongest known force for evolutionary selection in the recent history of the human genome*” [4]. Severe malaria anaemia (SMA) is a well-recognized syndrome of falciparum malaria [3, 5]. The concentration of haemoglobin in children with SMA is below 5 g/dL and patients exhibit weakness, prostration, tachycardia and respiratory distress. This depth of anaemia can result in insufficient delivery of oxygen to tissues with resulting systemic lactic acidosis [5]. Lactic acidosis in malaria, if not corrected, is highly correlated with death [6].

Approximately 50% of Ugandan children with SMA have elevated blood lactate levels [3, 5, 6]. Blood lactate production results from host metabolism and has been found not to correlate with parasite burden. Although it has been shown that lactic acidosis can result from severe anaemia with inadequate tissue oxygenation, and that lactic acidosis is relieved by transfusion of red blood cells with restoration of tissue oxygenation [5], there is no recognized explanation for the observation that some children develop lactic acidosis with SMA while others do not. Because oxygen-dependent bioenergetics occurs within mitochondria and relies in part on proteins encoded by mitochondrial genes, this study was designed to explore whether genetic differences in host mitochondria might account for differences in tolerance to the decreased tissue oxygenation that accompanies SMA. To investigate whether or not mitochondrial sequence variation might be associated with the development of lactic acidosis in severe anaemia, a matched case–control study among children with SMA was performed to compare the entire mitochondrial genome among severely anemic children with and without lactic acidosis.

## Methods

### Patient selection

Patient blood samples for mitochondrial DNA sequencing were collected during the course of two prior studies [3, 5]. All samples were obtained from children aged 6 months to 5 years presenting for care to the paediatric acute care unit of Mulago Hospital in Kampala, Uganda. The hospital serves an indigent local community population which is ancestrally homogeneous with over 75% of mothers belonging to one of three tribes (Muganda 63%, Musoga 8%, and Munyankole 9%). All samples came from children with confirmed *P. falciparum* infection and with haemoglobin values ≤ 5 g/dL.

Blood lactate and haemoglobin levels were measured using point-of-care devices (LactatePro, Arkray; Hemocue 201) at the time of sample collection and prior to blood transfusion. Samples were frozen immediately after phlebotomy and stored in cryovials at − 20 **°**C or colder prior to testing. All samples were collected and stored with informed consent of the parent or guardian. Approval for sample collection, material transfer, and DNA sequencing was provided by the research ethics committee of Makerere University in Kampala, Uganda and by the Uganda National Council of Science and Technology.

From the above archive of frozen samples, two groups were selected based on the blood lactate level measured at the time of original phlebotomy: one group (n = 50) with blood lactate levels > 10 mM and one group with blood lactate levels < 5 mM (n = 50). Samples from the two groups were deliberately matched for patient age, gender, and haemoglobin concentration. Only patients who tested negative for the presence of haemoglobin S were included.

### DNA extraction

For each patient, DNA was purified from a frozen blood sample using the Autopure LS instrument following the manufacturer’s protocol (AutoGen, MA, USA). An additional purification was performed using the ZR-96 DNA Clean & Concentrator-5 kit (Zymo Research, CA, USA) to reduce heparin contamination. Purified DNA samples were then quantified using the Quant-iT PicoGreen dsDNA assay kit (Invitrogen, CA, USA) and measurements were made on an ultraviolet spectrophotometer (Gemini XPS).

### DNA library preparation

Library preparation for each DNA sample was performed by long-range PCR followed by Nextera XT (Illumina, CA, USA) as described in the Human mtDNA Genome protocol for the Illumina Sequencing Platform [7]. Briefly, two long PCR amplicons were generated using two sets of primers (MTL-F1 and MTL-R1, MTL-F2, and MTL-R2) designed to encompass the entire human mitochondrial genome. The first set of primers generated a 9.1 kb fragment using primers MTL-F1 (5′-AAA GCA CAT ACC AAG GCC AC-3′) and MTL-F2 (5′-TTG GCT CTC CTT GCA AAG TT-3′). The second set of primers generated an 11.2 kb fragment using primers MTL-F2 (5′-TAT CCG CCA TCC CAT ACA TT-3′) and MTL-R2 (5′-AAT GTT GAG CCG TAG ATG CC-3′). For each patient DNA, two long-range PCR amplifications were performed using the TaKaRa LA TaqDNA Polymerase kit with a DNA input of 20 ng. Post-PCR products were quantified as described above and fragment sizes were verified using the DNA 12000 Bioanalyzer kit (Agilent, CA, USA).

The two amplicons for each patient were pooled and libraries were generated using the NexteraXT protocol (Illumina, CA, USA) automated on the Sciclone (Caliper/Perkin Elmer) with a DNA input of 1 ng. The NexteraXT library prep kit is a tagmentation process that fragments and adds adaptors to those fragments using an engineered transposome. The tagmented DNA is then amplified using a limited-cycle PCR program which adds Illumina adapters and unique indexes for each patient amplicon pool. After PCR, samples are purified using Ampure XP beads (Beckman Coulter, MA, USA).

Final libraries were quantified and fragment sizes were determined using High Sensitive D1000 Screentapes on the TapeStation instrument (Agilent, CA, USA). The expected size of final libraries is in the range of 500–1000 bp. Accurate measurement of sequence ready libraries was performed using the KAPA Library Quantification Kit (KAPA Biosystems, MA, USA) and run on the 7500 Fast Real-Time PCR System (Applied Biosystems, CA, USA).

### Sequencing

The uniquely indexed patient libraries were pooled at equimolar concentrations based upon KAPA assay measurements, and the final pool was loaded at 10 pM (with 5% PhiX spike-in) on the Miseq instrument (Illumina, CA, USA) using a 600 cycle v3 Miseq kit (Illumina) to generate 300 bp paired end sequenced reads.

### Variant calling and coverage

Raw sequencing data were analysed using the BaseSpace mtDNA Variant Processor App (Illumina), a commercially available web-based analysis pipeline. Briefly, this tool performs alignment and variant calling of samples against a reference mitochondrial DNA (mtDNA) genome, generating BAM alignment files and variant call files (VCF) for each of the samples. The analysis was restricted to samples with > 90% coverage of the entire mitochondrial sequence at 10× reads or greater. Additional information on software components of this analysis pipeline can be found online (https://support.illumina.com/downloads/basespace-mtdna-variant-processor-app.html).

### Variant annotations

Variant annotations were performed using MSeqDR: the Mitochondrial Disease Sequence Data Resource Consortium’s tool called MvTool and VariantOneStop (https://mseqdr.org/). Briefly, VCF files were processed to the required format by using python scripts and run through mvTool to get variant annotations in csv format. mvTool converts dozens of mtDNA variant formats into a standard revised Cambridge Reference Sequence (rCRS)-based HGVS format.

In addition, annotations generated by VariantOneStop include multiple-population frequencies from Mitomap and HmtDB, as well as annotations from Ensembl VEP, Mutalyzer, ClinVar, and from the MSeqDR database. Functional predictions for missense variants were curated from MitImpact2 database which provides pre-computed pathogenicity predictions for mitochondrial variants (http://mitimpact.css-mendel.it/) and includes predictions from MToolBox, PolyPhen2, MutationVariant Taster, and SIFT tools.

Haplogroup assignment for each individual was performed using the mtDNA-Server analysis software (https://mtdna-server.uibk.ac.at/index.html), which utilizes the HaploGrep tool to determine haplogroup of mtDNA profiles (http://haplogrep.uibk.ac.at/). Bam files for each sample were uploaded directly into mtDNA-Server for this analysis.

### Statistical analysis

For each group the median number of variants per person relative to the Cambridge Reference Sequence was compared using the Wilcoxon test; and the average number of variants per 1000 nucleotides was compared using the Student’s t-test. The Fisher’s test was used to compare the frequency of disruptive non-synonymous variants in the NADH dehydrogenase gene cluster. An alpha value of 0.05 was used to assess statistical significance. No corrections were made for multiple comparisons.

## Results

From the original set of 100 archived samples, 7 were eliminated due to low DNA isolation and an additional 20 were eliminated due to low coverage after sequencing, leaving 73 subjects (36 with high blood lactate levels and 37 with normal lactates) for final analysis. See “Methods” for details. Clinical characteristics of the comparison groups are shown in Table 1. All patients had active *P. falciparum* malaria and met standard criteria for SMA. The two groups were well-matched for age and sex. Of particular importance, the two groups were matched for haemoglobin concentration and oxygen saturation, determinants of oxygen delivery, but had blood lactate levels that differed by more than fivefold.

**Table 1:** Clinical characteristics of the high lactate and normal lactate groups

	High lactate, n = 36	Normal lactate, n = 37
Median (IQR) lactate, mM	14.4 (13.6–15.3)	2.7 (1.9–3.3)
Median (IQR) age, years	2.3 (1.5–3.8)	2.5 (1.7–4.2)
Gender (female:male)	16:20	14:23
Median (IQR) haemoglobin, g/dL	2.8 (2.6–3.4)	3.0 (2.8–3.7)
Mean (SD) O_2_-saturation, %	96.8 (4.1)	97.5 (2.4)
Mean (SD) log parasitaemia^a^	4.66 (0.79)	4.28 (1.07)

^a^The log_10_ of the number of parasitized red cells per μL

### Haplogroup assignments

Mitochondrial haplogroup assignments were made as described in the Methods. As expected in a population of individuals with common ancestry from East Africa, haplogroups L0–L4 were most commonly found. Because all subjects came from a common ancestral background, there was no difference in the distribution of haplogroups between the two groups see Table 2.

**Table 2:** Mitochondrial haplogroup assignments in 73 Ugandan patients with severe malaria anaemia

	L0	L1	L2	L3	L4	L5	Other	Total
High blood lactate	9	4	6	14	3	0	0	36
Normal blood lactate	8	3	6	14	4	1	1	37
Total	17	7	12	28	7	1	1	73

The distribution of haplotypes is not different in the two groups

### Mitochondria DNA sequence variation compared with the Cambridge Reference Sequence

**Fig. 1:**
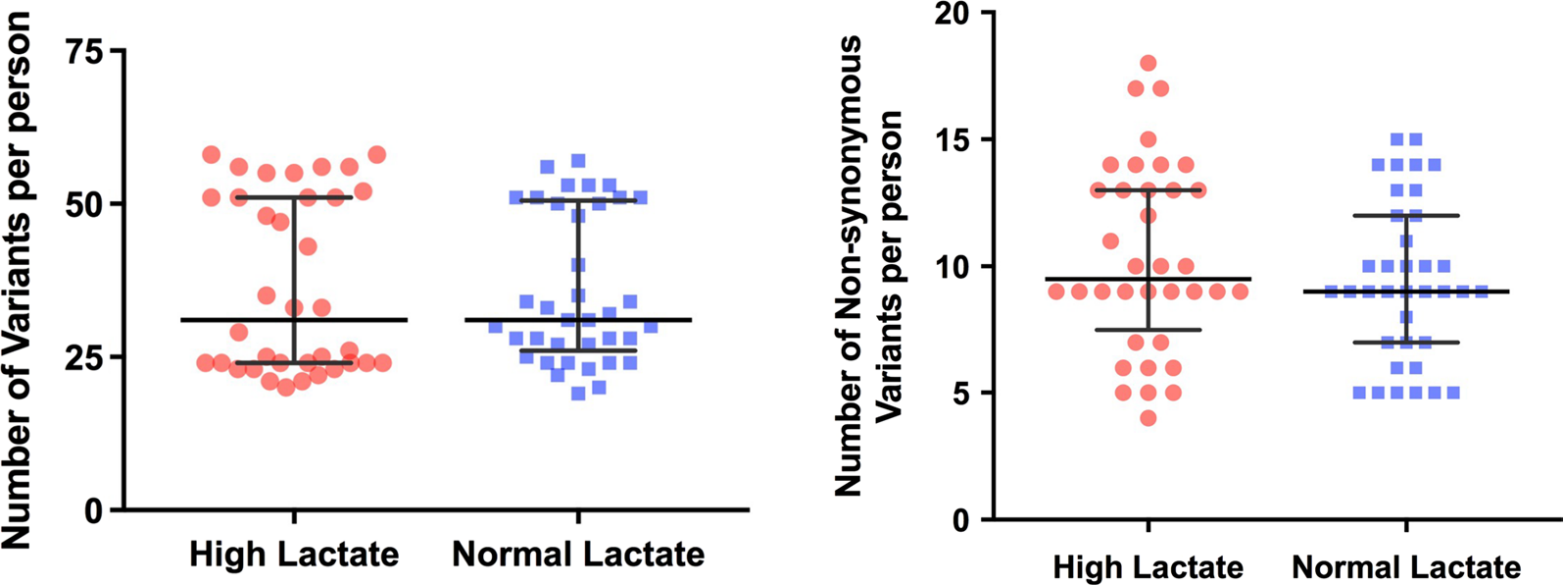
Distribution of mitochondrial gene nucleotide variants in oxidative phosphorylation genes. The number of variants per person relative to the revised Cambridge Reference Sequence over the region of mitochondria encoding genes for oxidative phosphorylation is shown. Data from patients with high blood lactate levels (n = 36) and normal blood lactate levels (n = 37) are shown. Variants found at locations outside the coding sequence for the oxidative phosphorylation proteins are not included. Left panel shows results for any form of sequence variant. Right panel shows non-synonymous variants only. Horizontal bars are medians and inter-quartile ranges

In the total group of 73 individuals tested, 4770 nucleotide variants were observed when compared with the revised Cambridge Reference Sequence resulting in an average frequency per individual of 3.97 variants per 1000 nucleotides (see Table 3). The median (IQR) number of variants/person for the entire mitochondrial sequence in the high lactate and normal lactate group was 57.5 (42–93) and 61 (45–90), respectively. The distribution of variants for the genes associated with oxidative phosphorylation was not statistically different between the two groups (see Fig. 1, left panel). Most of the variants occurred in more than one individual, and thus the number of unique variants identified in the entire cohort was 665 variants. The majority of these (565/665) were synonymous variants, while the remaining variants were non-synonymous variants expected to result in missense changes at the amino acid level. In-frame indels and loss-of-function (nonsense, frameshift) variants were not identified.

**Table 3:** Mitochondrial gene variants in 73 children with severe malaria anaemia

	Any variant (full sequence)	Any variant (oxphos genes)	Non-synonymous (oxphos genes)
Number of variants found			
All subjects, n = 73	4770	2640	722
High lactate, n = 36	2365	1315	373
Normal lactate, n = 37	2405	1325	349
Median (IQR) variants/patient			
All subjects, n = 73	60 (42–91)	31 (24–51)	9 (7–13)
High lactate, n = 36	57.5 (42–93)	31 (24–51)	9.5 (8.5–13)
Normal lactate, n = 37	61 (45–90)	31 (27–50)	9 (7–12)
Average (SD) variants/1000 nucleotides			
All subjects, n = 73	3.97 (1.42)	3.19 (1.16)	0.87 (0.30)
High lactate, n = 36	3.99 (1.53)	3.22 (1.26)	0.91 (0.33)
Normal lactate, n = 37	3.95 (1.32)	3.16 (1.07)	0.83 (0.26)

The number of variants, the median number (inter-quartile range, IQR) of variants per patient, and the average number of variants per patient per 1000 nucleotides is shown. The average number of non-synonymous variants per patient per 1000 nucleotides in genes of oxidative phosphorylation (oxphos) is not significantly different between the high lactate and normal lactate group, p = 0.08 (t-test)

### Non-synonymous variants within oxidative phosphorylation genes

Although most variants resulted in synonymous coding, non-synonymous variants in the regions between nucleotide 3307 and 15,887 that code for 13 genes responsible for oxidative phosphorylation were the main focus of analysis as these variants are capable of creating mutations affecting enzyme function. Non-synonymous variants to the revised Cambridge Reference Sequence were identified using the MSeqDR annotations. When combining the results for all oxidative phosphorylation genes, the frequency of non-synonymous variants per person was not different between the groups with high and normal lactate levels (Fig. 1, right panel). Each of the oxidative phosphorylation genes was then analysed separately (see Table 4). Although the frequency of non-synonymous variants per person per 1000 nucleotides was slightly higher in the high lactate group for nearly every gene, the differences were not statistically significant. In both patient groups, non-synonymous variants per patient per 1000 nucleotides were found less frequently in ND4L/4 and were found more frequently in ATP8/6, ND3, and Cyto-b compared with the other oxidative phosphorylation genes. The number of individuals with one or more non-synonymous variants in each of the oxidative phosphorylation genes was also not different between the two groups.

**Table 4:** Non-synonymous variants per person compared with the Cambridge Reference Sequence

	All subjects n = 73		High lactate n = 36			Normal lactate n = 37		
	Variants/person	Variants/person/1000 nts	Variants/person	Variants/person/1000 nts	Number of individuals	Variants/person	Variants/person/1000 nts	Number of individuals
ND1	0.21	0.21	0.25	0.26	8	0.16	0.17	6
ND2	0.49	0.47	0.56	0.53	12	0.43	0.42	11
COI	0.60	0.39	0.61	0.40	17	0.59	0.39	13
COII	0.11	0.16	0.14	0.20	4	0.08	0.12	3
ATP8/6	2.44	2.90	2.42	2.87	36	2.46	2.92	37
COIII	0.15	0.19	0.17	0.21	6	0.14	0.17	6
ND3	1.23	3.56	1.33	3.85	36	1.14	3.28	24
ND4L/4	0.18	0.11	0.19	0.12	7	0.16	0.10	4
ND5	1.38	0.76	1.56	0.86	25	1.22	0.67	24
ND6	0.14	0.26	0.17	0.32	5	0.11	0.21	4
Cyto-b	2.96	2.59	2.97	2.60	36	2.95	2.58	37
Total	9.89	0.87	10.36	0.91	36	9.43	0.83	37

The average number of non-synonymous variants per person and the average number of variants per person per 1000 nucleotides (nts) is shown. The number of individuals with at least one non-synonymous variant is shown

**Fig. 2:**
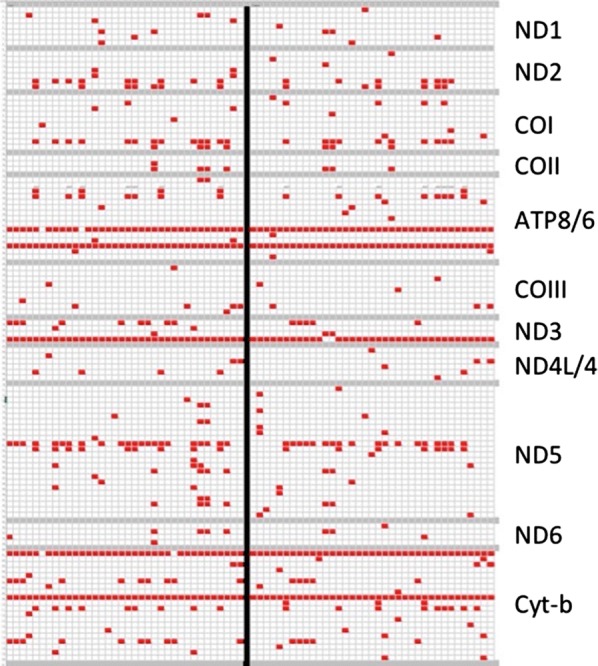
Non-synonymous variants in oxidative phosphorylation genes among patients with and without elevated blood lactate levels. Patients with high blood lactate (n = 36) are on the left of the solid black line; patients with normal lactate levels (n = 37) are on the right. Each patient is represented by a column and each variant location by a row. Non-synonymous variants are shown as red squares. To simplify the figure, nucleotide positions (rows) without variants are not shown

Non-synonymous variants when present were seen in as few as 0.3% to as many as 100% of individuals depending on the nucleotide position. The distribution of individual non-synonymous variants in the study population is shown in Fig. 2.

### Non-synonymous variants predicted to represent a disruptive variant

Because non-synonymous variants are predicted to result in different degrees of disruption of the final protein, the analysis further focused on those variants predicted to be most disruptive to the oxidative phosphorylation polypeptides. Four different software applications designed to predict disruptive variants were used: MToolBox [8, 9], PolyPhen2 [10], SIFT [11], and Mutation Taster [12]. Of the 100 different non-synonymous variants found within genes coding for oxidative phosphorylation, the 15 variants with the highest MToolBox disruption scores are shown in Table 5. Half of these occurred within genes coding for NADH dehydrogenase subunits (ND genes). When the data for the ND genes were combined in post hoc analysis, the frequency of disruptive variants at the seven ND locations was 0.087 in the high lactate group and 0.046 in the normal lactate group. Although this difference was not statistically significant (p = 0.052, Fisher’s test) it suggests that disruptive variants of ND genes may be a target area for future study.

**Table 5:** Non-synonymous variants predicted to disrupt gene products associated with oxidative phosphorylation

Position	Variant	Gene	Amino acid variant	MToolBox score	PolyPhen2 score	Mutation taster	SIFT score	High lactate	Normal lactate	MitoMap frequency^a^
7080	T>C	COI	Phe393Leu	0.695	0.99	0.99	0.16	0	1	1
8027	G>A	COII	Ala148Thr	0.635	1.00	1.00	0.61	4	3	477
8417	C>T	ATP8	Leu18Phe	0.713	0.99	1.00	0.45	2	0	29
8460	A>G	ATP8	Asn32Ser	0.627	0.97	1.00	0.50	4	1	182
8582	C>T	ATP6	Ala19Val	0.697	1.00	1.00	0.04	0	2	29
8668	T>C	ATP6	Trp48Arg	0.816	1.00	1.00	0.34	0	1	12
9055	G>A	ATP6	Ala177Thr	0.533	0.51	1.00	0.55	1	1	9
10,086	A>G	ND3	Asn10Asp	0.594	0.90	1.00	0.02	9	5	160
11,172	A>G	ND4	Asn138Ser	0.769	0.03	1.00	0.41	4	1	182
12,092	C>A	ND4	Leu445Ile	0.666	0.02	1.00	0.40	0	1	5
12,814	G>A	ND5	Ala160Thr	0.688	0.73	1.00	0.39	0	1	2
13,651	A>G	ND5	Thr439Ala	0.690	0.08	1.00	0.52	1	0	51
13,789	T>C	ND5	Tyr485His	0.714	0.17	1.00	0.50	4	2	720
14,000	T>A	ND5	Leu555Gln	0.675	0.95	1.00	0.31	4	2	465
15,030	T>A	Cyto-b	Phe95Tyr	0.491	0.52	1.00	1.00	2	2	Novel

For each variant, the number of individuals with the variant in the high lactate and normal lactate groups is shown. The frequency of each variant in the MitoMap database for individuals with a predicted L0–L5 haplotype is also shown

^a^MitoMap frequency for the L0–L5 haplotypes. A total of 4025 individuals with a predicted L0–L5 haplotype are present in the Mitomap database (https://www.mitomap.org). For MToolBox, PolyPhen2, and Mutation Taster a higher score predicts a disruptive variant. For SIFT, a lower score predicts a disruptive variant

## Discussion

*Plasmodium falciparum* malaria is a highly lethal and common disease of children worldwide. Lactic acidosis is a well-established complication of malaria infection and is associated with an increased risk for fatal outcome [6]. While lactic acidosis is present in approximately half of those with SMA, individual patients exhibit different levels of lactic acidosis despite the same degree of anaemia and many patients tolerate severe anaemia without developing elevated blood lactate levels [3, 5, 6]. The extent to which such differences are related to mitochondrial gene polymorphisms is unknown and was the focus of this study.

Malaria exerts a strong evolutionary selection pressure and is known to be associated with a large number of nuclear gene adaptations [4]. Although malaria infection has been associated with host mitochondrial pathology [13], previous studies exploring an association between severe malaria syndromes and human mitochondria DNA sequence variations were not identified. In this study, mitochondrial gene sequences between a cohort of children with or without lactic acidosis in the setting of SMA were compared for the first time. The two patient groups were well matched for ethnic background, and patients lived in close proximity to the hospital. The majority of mothers belonged to just three similar tribal affiliations. Mitochondrial haplotype analysis was balanced in the two groups and reflected haplogroups expected in an East African population [14, 15]. Given the genetic heritage of the participants, a large number of synonymous and non-synonymous genetic variants were found when compared with the Cambridge Reference Sequence. These variants occurred throughout the mitochondrial genome.

The hypothesis of this study was that mitochondrial gene sequences might differ between those individuals who developed high blood lactate levels at the time of severe anaemia and those who did not. In particular, mitochondrial genes directly involved in oxidative phosphorylation were analyzed. Although the comparisons did not reach statistical significance for the sample size tested, the average number of non-synonymous variants per individual, the number of individuals with non-synonymous variants, and the occurrence of variants predicted to cause protein disruption were all higher in the group with elevated blood lactate levels. In particular, non-synonymous variants predictive to disrupt at least one member of the NADH dehydrogenase gene cluster were twice as common in children with high lactates, a finding that did not quite reach statistical significance (p = 0.052). Future studies with a larger sample size are needed to verify whether or not NADH dehydrogenase mutations might contribute to intolerance to severe anaemia.

The findings of this study suggest that the determinants of high blood lactate levels in patients with SMA may be related either to gene polymorphisms outside the mitochondrial genome or to other factors. Because the great majority of mitochondrial proteins are encoded by nuclear genes [16], variations in those genes might influence mitochondrial function. Alternatively, variation in other nuclear genes such as those of the glycolytic pathway, the Krebs cycle, or those affecting NAD+/NADH levels may influence blood lactate levels during severe anaemia. In addition, lactic acidosis in SMA may also be related to factors unrelated to host genetics such as microvascular physiology, nutritional status, or duration of illness. Understanding the fundamental drivers for life-threatening lactic acidosis in malaria awaits further research.

This study has both strengths and weaknesses. The comparison groups were homogeneous and well matched for clinical features including age, gender, and the degree of anaemia and haemoglobin oxygen saturation. In addition, all subjects were documented to be negative for the sickle haemoglobin gene which would be the most likely confounding variable to affect comparisons. The method of sample preparation avoided confounding from parasite mitochondrial DNA. However, the two groups were not compared for variations in nuclear genes that affect mitochondrial function, nor were the groups compared for the number of mitochondria present in tissues expected to produce the greatest amount of lactate. In addition, the number of individuals tested was insufficient to detect low frequency differences in mitochondrial gene sequences between the comparison groups.

## Conclusion

In this prospective case–control investigation of 73 Ugandan children with severe anaemia and *P. falciparum* malaria, mitochondria sequence variations were observed to occur in approximately 4/1000 nucleotides compared with the Cambridge Reference Sequence. However, within the constraints of the sample size tested, no statistically significant difference in mitochondrial DNA sequence variation was observed when comparing those with and without lactic acidosis. Increased blood lactate, an important marker for decreased survival in SMA, does not appear to be strongly associated with a high prevalence host mitochondrial DNA sequence variation.

## Abbreviation

SMA: severe malaria anaemia
